# Myocardial Bridging in Patients Undergoing Coronary Angiography for Coronary Artery Disease

**DOI:** 10.7759/cureus.60087

**Published:** 2024-05-11

**Authors:** Ajay Mishra, Shahnawaz Jafri, Saboor Mateen, Firdaus Jabeen, Irshad Wani

**Affiliations:** 1 Internal Medicine, Era’s Lucknow Medical College and Hospital, Lucknow, IND; 2 Cardiology, Krishna Institute of Medical Sciences, Hyderabad, IND; 3 Internal Medicine, Era's Lucknow Medical College and Hospital, Lucknow, IND; 4 Cardiology, Career Institute of Medical Sciences & Hospital, Lucknow, IND

**Keywords:** myocardial ischemia, cardiac chest pain, coronary angiography, coronary artery disease, myocardial bridging

## Abstract

Introduction

Myocardial bridge is a rare, benign, normal anatomical variant of the coronary artery that puts the patient at risk for significant cardiac symptoms, resulting in myocardial ischemia, arrhythmia, and sudden cardiac death. The aim of the study was to assess the prevalence and characteristics of myocardial bridging (MB) in patients with chest pain undergoing coronary angiography.

Methodology

A total of 1301 patients presenting with chest pain suggestive of acute coronary syndrome with associated non-invasive supportive cardiac evaluation were subjected to coronary angiography by Philips Allura Xper FD10 Cath Lab (Philips Healthcare, Andover, MA) and evaluated.

Results

Out of 1301 patients, the mean age was 54.70 ± 11.41 years with a male-to-female ratio of 1.9:1. Tobacco use and diabetes mellitus were the most common associated risk factors (49% and 44%, respectively). MB was seen in 51 patients, making the prevalence 3.9%, with male predominance over females in the ratio of 3.9:1. The most common clinical presentation was unstable angina (UA) (n = 22, 43.1%), followed by stable angina (SA) (n = 11, 21.6%), non-ST-elevation myocardial infarction (NSTEMI) (n = 10, 19.6%), and ST-elevation myocardial infarction (STEMI) (n = 8, 15.7%). Myocardial bridges were more common among patients with stable coronary artery disease. The left anterior descending artery (n = 51, 3.9%) was involved in all the cases and the middle segment was affected in all patients with MB. Among patients with myocardial bridge, 26 patients (51%) had atherosclerosis and 25 patients had a normal artery. Among patients with myocardial bridge with atherosclerosis, 17 patients (65%) had atherosclerosis in the same artery in which the myocardial bridge was present. Among patients with myocardial bridge with atherosclerosis, nine patients (52%) had atherosclerosis proximal to the bridge, three patients (17%) had atherosclerosis distal to the bridge, and five patients (31%) had atherosclerosis both proximal and distal to the bridge.

Conclusion

The prevalence of MB in the Indian population is significantly lower than in the Western populations, and it is significantly higher in the male population with patients diagnosed as normal coronaries on coronary angiography.

## Introduction

The phenomenon known as myocardial bridging (MB) is marked by the overlying myocardial tissue compressing a tunneled coronary artery during diastole, which thereafter totally disappears [1]. Although this bridging is usually not harmful, it can be linked to a number of cardiac events, including myocardial infarction, arrhythmia, and unexpected death [2]. Left ventricular hypertrophy, microvascular dysfunction, coronary vasospasm, or diastolic dysfunction can cause signs in previously asymptomatic patients [3].

Most series of myocardial bridges reveal a male predominance [4]. The prevalence reported has been variable, ranging from 0.5% to 86% among diverse investigations, with autopsy series reporting a higher prevalence than angiographic studies [5]. The middle section of the left anterior descending (LAD) coronary artery is the most recurrent site, accounting for 18% and 40% of cases. If isolated myocardial bridges are found and treated, their long-term prognosis is acceptable [6].

There are two types of MB: single and multiple. The latter might occur in the same coronary artery or one of its branches [7]. Moreover, it can be separated into two categories: deep and superficial muscle kinds. The coronary flow is not hindered by the superficial form during systole, but the deep form has the potential to compress the coronary artery, reduce the flow, and result in myocardial ischemia [4]. Class 1 (systolic coronary narrowing <50%), class 2 (systolic coronary constriction of 50-75%), and class 3 (systolic coronary narrowing beyond 75%) are the three categories of systolic coronary narrowing [8]. Anatomical characteristics vary widely, measuring from 2.3 and 42.8 mm in length, 1.0 and 3.8 mm in thickness, and 5° to 90° in angle between the long axis of the muscle fibers and the long axis of the crossing vessel [9]. According to several studies, the bridges' average length was 14.64 ± 9.03 mm, and their average thickness was 1.23 ± 1.32 mm [10].

The real incidence of MB in various populations is not entirely understood and varies greatly depending on the techniques employed to identify this anatomic anomaly. As such, it is challenging to determine the precise prevalence of myocardial bridges. There have been several necropsy series conducted, with MB rates ranging from 5% to 86% recorded [7]. On average, one-fourth of individuals may have MBs. Compared to coronary angiography, which often locates the bridges with clear systolic compression (milking effect), the prevalence of MBs was higher in pathological series, which included thin MBs or even myocardial strands with negligible hemodynamic effects. As a result, the angiographic detection rate of MBs ranges from 0.5% to 12% [5] when the patient is at rest to 40% when nitroglycerin is injected intracoronarily.

Numerous factors have been identified as the cause of the disparity between the rates of tunneled arteries reported at necropsy and angiographic measurements. The MB's length and thickness, the coronary artery and myocardial fibers' reciprocal orientation, the existence of loose connective or adipose tissue encompassing the bridged segment, the existence of an aortic outflow tract obstruction (where the MB's systolic tension overcomes the intracoronary artery pressure), the intrinsic tone of the coronary artery wall, the existence of a proximal coronary fixed obstruction (which lowers the distal intracoronary pressure), the state of the heart rate and myocardial contractility at the time of the angiography, and researcher experience are some of these factors [11].

Because intravascular ultrasonography (IVUS) is becoming more widely used and is more sensitive to identifying moderate compression, the occurrence of MB has been recorded to reach up to 23% [12]. The more current technology known as cardiac computed tomography (CCT) has drastically raised the rate of detection of MB (in vivo) even in situations when the milking effect and/or alterations in artery course at conventional angiography are minimal or nonexistent. CCT's multi-plane and three-dimensional capabilities have made this possible. As a result, the CCT-based occurrence of MBs increases to 5%-76%, dependent upon the study population's inherent heterogeneity, the types of scanners used, and the MB pattern (deep versus superficial encasement) [5,11].

The investigation's purpose was to determine the frequency and characteristics of MB in patients who had chest pain that might have been a sign of coronary artery disease (CAD) and were having coronary angiography.

## Materials and methods

The study was performed over a two-year period in a north Indian tertiary care hospital addressing a semi-urban population. There were 1301 instances in all that had coronary artery disease at the time of presentation.

Sample size

Considering an estimated 3.17% prevalence of MB [13], an acceptable error of 20% for detecting results with 80% research power, and a 10% data loss, 1300 was the computed size of the sample.

Inclusion criteria

All patients above 18 years of age of either gender presenting with typical anginal pain/cardiac/likely cardiac (pressure, tightness, squeezing, heaviness, or burning) or non-cardiac chest pain were considered for angiography if they showed signs of ST-elevation myocardial infarction (STEMI) with or without positive biomarkers, non-ST-elevation myocardial infarction (NSTEMI) with or without positive biomarkers (unstable angina), ST-segment depression with or without elevated biomarkers (unstable angina), positive treadmill test, or positive biomarkers within six hours of chest pain onset with a possible cardiac origin only. The patients underwent angiography and the findings were noted and analyzed.

Exclusion criteria

Patients with acute/chronic renal failure, severe anemia (hemoglobin < 7 mg/dl), valvular heart disease, pregnancy, or hypersensitivity to contrast used in coronary angiography were not included in the investigation. Informed consent and written consent from all the research subjects regarding participants/procedure/effects (short/long term) in the study were taken.

Methods of collection of data

In each case, a detailed history/examination related to the presenting symptoms and possible risk factors was taken. Hematological, biochemical, and cardiac workups, including electrocardiogram (ECG), 2D echocardiography (2D Echo), stress test, and biomarkers, were done. Quantitative coronary angiography was done to describe the MB segment. The patients underwent coronary angiography on Philips Allura Xper FD10 Cath Lab (Philips Healthcare, Andover, MA).

Statistics

Chi-square and ANOVA (analysis of variance) were used in the statistical analysis, which was performed employing SPSS version 15.0 (SPSS Inc., Chicago, IL) statistical analysis software.

## Results

A total of 1301 patients were enrolled in the study who underwent coronary angiography, of which 853 (65.6%) were males and 448 (34.4%) were females. The male-to-female ratio in this study was 1.9:1. The mean age of the patients was 54.70 ± 11.41 years (ranging from 18 to 92 years). The maximum number of patients were in the age group of 60 to 70 years (n = 378, 29.1%), followed by the age group of 50 to 60 years (n = 373, 28.7%), thereby indicating that more than half of the population (57.5%) was in the age bracket of 50-70 years (Table 1). Tobacco use and diabetes were the most common associated risk factors in patients overall with nearly half the population consuming tobacco in some form (49%), followed by diabetes (44%) (Table 1).

**Table 1:** Demographic profile of the study population

Age group	Total	
	Number (n = 1301)	Percentage (%)
Gender distribution		
Males	853	65.6%
Females	448	34.4%
Age distribution		
≥18 and <20 years	2	0.2%
20-30 years	16	1.2%
30-40 years	87	6.7%
40-50 years	308	23.7%
50-60 years	373	28.7%
60-70 years	378	29.1%
≥70 years	137	10.5%
Co-morbidities		
Tobacco	648	49%
Diabetes	573	44%

Among 1301 patients, the maximum number of patients had triple vessel disease (n = 292, 22.4%), followed by double vessel disease (DVD) (n = 274, 21.1%) and single vessel disease (SVD) (n = 209, 16.1%) (Table 2).

**Table 2:** Primary finding of coronary arteries on coronary angiography

Diagnosis	Total	
	Number (n = 1301)	Percentage (%)
Single vessel disease	209	16.1%
Double vessel disease	274	21.1%
Triple vessel disease	292	22.4%
Insignificant coronary artery disease	50	3.8%
Normal coronary arteries	476	36.6%

MB was observed in 51 patients, making the occurrence of MB in our study 3.9% (Table 3).

**Table 3:** Prevalence of myocardial bridging among total patients (n = 1301)

Total cases of myocardial bridging		51
Prevalence (%)		3.9%
Standard error of prevalence		0.54
95% confidence interval of prevalence	Lower	2.87
	Upper	4.97

Among patients with a myocardial bridge (n = 51), the majority of patients were males (n = 40, 4.7%) and the male-to-female ratio was 3.9:1, which was statistically significant (p < 0.05). The maximum number of patients was in the age group of 50 to 60 years (n = 16) and 60 to 70 years (n = 16), followed by the age group of 40 to 50 years (n = 15). However, no significant difference was observed in the proportion of myocardial bridges between the age groups (p = 0.600). Among patients with myocardial bridge, tobacco use (n = 32, 4.9%) was the most common cardiovascular risk factor, followed by diabetes mellitus (n = 16, 2.8%). We observed no significant association between myocardial bridge and comorbidities or history of coronary artery disease (Table 4).

**Table 4:** Association of myocardial bridging with different variables

Variables	Total number (n = 1301)	Myocardial bridging		Chi-square	p-value
		Number (n = 51)	Percentage (%)		
Gender distribution					
Males	853	40	4.7%	3.89	0.049
Females	448	11	2.5%		
Age groups					
≥18 and <20 years	2	0	0%	4.57	0.600
20-30 years	16	0	0%		
30-40 years	87	1	1.1%		
40-50 years	308	15	4.9%		
50-60 years	373	16	4.3%		
60-70 years	378	16	4.2%		
≥70 years	137	3	2.2%		
Co-morbidities, habits, and family history					
Hypertension	513	23	4.50%	0.71	0.398
Diabetes	573	16	2.80%	3.46	0.063
Tobacco use	648	32	4.90%	3.55	0.059
Family history of coronary artery disease	298	7	2.30%	2.53	0.111

Unstable angina (UA) (n = 22, 43.1%), stable angina (SA) (n = 11, 21.6%), and NSTEMI (n = 10, 19.6%) were the most frequent clinical presentations, followed by STEMI (n = 8, 15.7%) in individuals with MB-related conditions. The difference in proportion for various clinical presentations was observed to be significant (p = 0.017) (Table 5). While exploring coronary arteries with a coronary angiogram, normal coronaries were found in 30 patients (58.5%), followed by single, double, and triple vessel disease in decreasing order (23.5% vs. 17.6% vs. 0), a difference that was statistically significant.

**Table 5:** Association of myocardial bridging with clinical presentation and diagnosis STEMI: ST-elevation myocardial infarction; NSTEMI: non-ST-elevation myocardial infarction; CAD: coronary artery disease.

Variables	Myocardial bridging		Chi-square	p-value
	Number (n = 51)	Percentage (%)		
Clinical presentation				
Stable angina	11	21.6%	9.31	0.03
Unstable angina	22	43.1%		
NSTEMI	10	19.6%		
STEMI	8	15.7%		
Diagnosis				
Insignificant CAD/normal coronaries	30	58.8%	37.24	<0.001
Single vessel disease	12	23.5%		
Double vessel disease	9	17.6		
Triple vessel disease	0	0%		

The left anterior descending artery (n = 51, 3.9%) was involved in all the cases and the middle segment was affected in all patients with MB (Table 6).

**Table 6:** Vessel and site of myocardial bridging (n = 51)

Component		Number	Percentage (%)
Vessel	Left anterior descending artery	51	3.9%
Site	Middle segment	51	3.9%

The average length of the myocardial bridge was 10.79 ± 5.08 mm (range: 4.13-27.10 mm) and the mean diameter in systole of MB was found to be 2.09 ± 0.95 mm (range: 1.00-6.23 mm) (Table 7).

**Table 7:** Length and diameter of myocardial bridges

Myocardial bridging parameter	Length (in millimeters)	Diameter in systole (in millimeters)
Mean	10.79	2.09
Standard deviation	5.08	0.95
Lower 95% CI	9.40	1.83
Upper 95% CI	12.19	2.35
Minimum	4.13	1.00
Maximum	27.10	6.23

Among patients with myocardial bridge, 26 patients (51%) had atherosclerosis, and 25 patients had normal arteries. Among patients with myocardial bridge with atherosclerosis, 17 patients (65%) had atherosclerosis in the same artery in which a myocardial bridge was present. Among patients with myocardial bridge with atherosclerosis, nine patients (52%) had atherosclerosis proximal to the bridge, three patients (17%) had atherosclerosis distal to the bridge, and five patients (31%) had atherosclerosis both proximal and distal to the bridge (Table 8).

**Table 8:** Myocardial bridge with atherosclerosis in different arteries

Component	Number of patients	Percentage (%)
Myocardial bridge with atherosclerosis and myocardial bridge with normal coronary artery		
Myocardial bridge with atherosclerosis	26	51%
Myocardial bridge with normal coronary artery	25	49%
Total	51	100%
Myocardial bridge with atherosclerosis in the same artery and myocardial bridge with atherosclerosis in the non-bridge artery		
Myocardial bridge with atherosclerosis in the same artery	17	65%
Myocardial bridge with atherosclerosis in the non-bridge artery	9	35%
Total	26	100%
Atherosclerosis in association with the site of myocardial bridge		
Atherosclerosis proximal to the bridge	9	52%
Atherosclerosis distal to the bridge	3	17%
Atherosclerosis both proximal and distal to the bridge	5	31%
Total	17	100%

## Discussion

A male-to-female ratio of 1.9:1 was found in the examined population of 1301 patients, with the majority of patients falling into the 60-70 years age range. This finding is consistent with research conducted in India by Sujatha et al. [13] wherein 2015 angiograms were examined and a male-to-female ratio of 2.5:1 was noted. Abhilash et al. [14] in another cross-sectional investigation of 10,492 cases reported a male-to-female ratio of 1.5:1. In a Western study, Jakub et al. [15] reported a male-to-female ratio of 1.6:1.

According to clinical diagnosis, we observed triple vessel disease in 292 (22.4%) patients and double vessel disease in 274 (21.1%). Normal coronary arteries were observed in 476 (36.6%) patients. However, Sujatha et al. [13] in their study reported a maximum number of patients as having SVD (472 patients, 23.4%), followed by normal coronary artery (456 patients, 22.6) patients and triple vessel disease (429 patients, 21.3%).

According to published research, Chinese and Japanese populations had the greatest rates of MB, i.e., 16% and 36%, respectively. In a cross-sectional investigation of 114 Japanese patients, Teragawa et al. [16] documented MB in 41 patients (36%) and found it in all of the patients' mid-segments of the LAD coronary artery. Qian et al. [17], in a cross-sectional investigation of 5525 patients in the Chinese population, reported MB in 888 patients angiographically with a prevalence of 16.1%. Mavi et al. [18] examined 7200 coronary angiographies in Turkish patients. In this investigation, 29 out of 7200 coronary angiographies (0.4%) had myocardial bridges. The location of the myocardial bridge was in the LAD coronary artery in 28 cases (96.5%), and the left circumflex coronary artery in one case (3.4%). Twenty-two (78.5%) of the total number of bridges on LAD were positioned on the middle segment, five (17.8%) on the distal segment, and one (3.5%) extended to both the middle and distal.

According to earlier research, the occurrence of MB ranges from 0.6% to 3.17% in South East Asia and India [19]. In a survey of 3200 patients, Harikrishnan et al. [20] found 21 (0.6%) patients to have MB. Additionally, they noted that in most patients, the proximal or mid-LAD coronary arteries contained myocardial bridges. In this study, the myocardial bridge's length ranged from 10 to 35 mm (mean: 24.5 ± 4.5 mm), while the percentage of diameter stenosis during systole varied from 40% to 90%.

Abhilash et al. [14], in a study from south India, analyzed 129 myocardial bridges (1.23%) in 10,492 coronary angiograms. The average length of myocardial bridges in this investigation was 24.53 mm. In further investigations, Sujatha et al. [13] observed 64 myocardial bridges (3.17%) in 2015 angiograms. Only the left coronary arteries LAD included all 64 of the myocardial bridges. Ninety-six patients (95.3%) had a single myocardial bridge, whereas three patients (4.7%) had two bridges. The central segment of the LAD contained the bulk of myocardial bridges (87.5%), with the distal (6.25%) and proximal (6.25%) parts of the LAD following closely behind.

In a study including 30 formalin-fixed cadaver hearts, Mahajan et al. [21] discovered myocardial bridges in 22 hearts that involved one or more major branches of the coronary arteries with more cases of left coronary artery (LCA) involvement than right coronary artery (RCA) involvement. The middle segment of the LAD artery was the site of the highest frequency of myocardial bridges. Matta et al. [22] examined 35,813 cases, out of which 510 had LAD myocardial bridge, taking the occurrence to 1.42%.

In our study, we observed a total of 51 myocardial bridges (3.9%) in 1301 angiograms. None of the patients in our study had myocardial bridges in more than one site. We also observed that in all the patients, the LAD in the middle segment was most commonly affected. Research by Jakub et al. [15] similarly found that those suffering from stable CAD had a higher prevalence of myocardial bridges. According to Sujatha et al. [13], the average length of myocardial bridges was 20.05 ± 12.61 mm. The range of diameter in systole was 1.00-6.23 mm. The mean diameter in the systole of myocardial bridges was 2.09 ± 0.950 mm. Ozlem et al. [23], in a cross-sectional investigation, studied 2547 patients, and the myocardial bridge was present in 26 patients. The average length of myocardial bridges was 10.7 ± 1.93 mm. The range of diameter in systole was 1.9-4.23 mm. The mean diameter in the systole of myocardial bridges was 1.09 ± 0.95 mm. In our study, in comparison to the previous studies, the range of length and diameter in the systole of myocardial bridges was 4.13-27.10 mm and 1.0-6.23 mm. The average length of myocardial bridges was 10.79 ± 5.08 mm. The average diameter in the systole of the myocardial bridge was 2.09 ± 0.95 mm.

In our study, out of 51 myocardial bridge patients, most patients (62.74%) had tobacco use as a cardiac risk factor. Hypertension was seen in 45.09% of patients and diabetes mellitus was observed in 31.37%. However, Jakub et al. [15] observed hypertension to be the most common (69.53%) cardiac risk factor. Diabetes mellitus was seen in 42% of patients and tobacco use in 14.68% of patients.

We observed that myocardial bridge was more common among patients with unstable angina followed by stable angina, NSTEMI, and STEMI. Among patients with myocardial bridge, 26 patients (51%) had atherosclerosis and 25 patients had normal arteries. Among patients with myocardial bridges with atherosclerosis, 17 patients (65%) had atherosclerosis in the same artery in which a myocardial bridge was present. Among patients with myocardial bridges with atherosclerosis, nine patients (52%) had atherosclerosis proximal to the bridge, three patients (17%) had atherosclerosis distal to the bridge, and five patients (31%) had atherosclerosis both proximal and distal to the bridge, which is in concurrence with previous studies from Indian and Western populations [14,15,20,24].

Jakub et al. [15] included 298,558 patients in their study, in which the myocardial bridge was present in 2425 patients (0.81%). In this study, myocardial bridges were more common among patients with stable angina (1066 patients, 43.96%) than unstable angina (873 patients, 36%), followed by STEMI (199 patients, 8.21%) and NSTEMI (186 patients, 7.67%).

Limitations

Further multi-centric studies with a larger study population will help to understand the true prevalence and associations with various demographic factors. Also, it is imperative to explore the genetic and molecular patterns of MB for early detection of the same.

## Conclusions

If detected early enough, myocardial bridge, a common undetected inborn cardiac defect, has a favorable prognosis. It needs to be taken into account, particularly in those with documented myocardial ischemia or angina-like chest pain who are at low risk for coronary atherosclerosis. The outcomes of the current examination demonstrated that the prevalence of MB in the Indian population is significantly lower than in Western populations and it is significantly higher in the male population with patients diagnosed as having normal coronaries on coronary angiography.

## References

[1] Akdemir R, Gunduz H, Emiroglu Y, Uyan C (2002). Myocardial bridging as a cause of acute myocardial infarction: a case report. BMC Cardiovasc Disord.

[2] Dursun I, Bahcivan M, Durna K, Ibrahimov F, Erk NH, Yasar E, Sahin M (2006). Treatment strategies in myocardial bridging: a case report. Cardiovasc Revasc Med.

[3] Corban MT, Hung OY, Eshtehardi P (2014). Myocardial bridging: contemporary understanding of pathophysiology with implications for diagnostic and therapeutic strategies. J Am Coll Cardiol.

[4] Ferreira AG Jr, Trotter SE, König B Jr, Décourt LV, Fox K, Olsen EG (1991). Myocardial bridges: morphological and functional aspects. Br Heart J.

[5] Lee MS, Chen CH (2015). Myocardial bridging: an up-to-date review. J Invasive Cardiol.

[6] Juilliére Y, Berder V, Suty-Selton C, Buffet P, Danchin N, Cherrier F (1995). Isolated myocardial bridges with angiographic milking of the left anterior descending coronary artery: a long-term follow-up study. Am Heart J.

[7] Möhlenkamp S, Hort W, Ge J, Erbel R (2002). Update on myocardial bridging. Circulation.

[8] Noble J, Bourassa MG, Petitclerc R, Dyrda I (1976). Myocardial bridging and milking effect of the left anterior descending coronary artery: normal variant or obstruction?. Am J Cardiol.

[9] Kosiński A, Grzybiak M (2001). Myocardial bridges in the human heart: morphological aspects. Folia Morphol (Warsz).

[10] Lujinović A, Kulenović A, Kapur E, Gojak R (2013). Morphological aspects of myocardial bridges. Bosn J Basic Med Sci.

[11] Alegria JR, Herrmann J, Holmes DR Jr, Lerman A, Rihal CS (2005). Myocardial bridging. Eur Heart J.

[12] Tsujita K, Maehara A, Mintz GS (2008). Comparison of angiographic and intravascular ultrasonic detection of myocardial bridging of the left anterior descending coronary artery. Am J Cardiol.

[13] Sujatha M, Devi VS, Raju CSS, Yugandhar B, Nagaraju Nagaraju (2015). Angiographic aspects of myocardial bridges. Int J Anat Res.

[14] Abhilash SP, Kumar KB, Velappan P, Gupata P, Viswanathan S, Koshy AG (2015). Myocardial bridging - clinical and angiographic profile in last 5 years; a study of 129 cases. Kerala Heart J.

[15] Podolec J, Wiewiórka Ł, Siudak Z (2018). Prevalence and clinical presentation of myocardial bridge on the basis of the National Polish Percutaneous Interventions Registry and the Classification of Rare Cardiovascular Diseases. Kardiol Pol.

[16] Teragawa H, Fukuda Y, Matsuda K (2003). Myocardial bridging increases the risk of coronary spasm. Clin Cardiol.

[17] Qian JY, Zhang F, Dong M (2009). Prevalence and characteristics of myocardial bridging in coronary angiogram--data from consecutive 5525 patients. Chin Med J (Engl).

[18] Mavi A, Sercelik A, Ayalp R, Karben Z, Batyraliev T, Gumusburun E (2008). The angiographic aspects of myocardial bridges in Turkish patients who have undergone coronary angiography. Ann Acad Med Singap.

[19] Singla SK, Singh R (2021). Proportion of myocardial bridge in a tertiary care hospital of North India: a retrospective observational study. J Clin Diagn Res.

[20] Harikrishnan S, Sunder KR, Tharakan J, Titus T, Bhat A, Sivasankaran S, Bimal F (1999). Clinical and angiographic profile and follow-up of myocardial bridges: a study of 21 cases. Indian Heart J.

[21] Mahajan A, Patil S, Vasudeva N (2017). Myocardial bridging: a study on the cadaveric hearts of the North Indian adult population. Nat J Clin Anat.

[22] Matta A, Canitrot R, Nader V (2021). Left anterior descending myocardial bridge: angiographic prevalence and its association to atherosclerosis. Indian Heart J.

[23] Soran O, Pamir G, Erol C, Kocakavak C, Sabah I (2000). The incidence and significance of myocardial bridge in a prospectively defined population of patients undergoing coronary angiography for chest pain. Tokai J Exp Clin Med.

[24] Javadrashid R, Tarzamni MK, Aslanabadi N, Ghaffari M, Salehi A, Sorteji K (2009). Myocardial bridging and coronary artery anomalies detected by ECG-gated 64-row multidetector computed tomography angiography in symptomatic patients. Folia Morphol (Warsz).

